# In Memoriam, Samuel David Gross

**Published:** 1884-07

**Authors:** 


### In Memoriam

WITHIN THIS URN LIE THE ASHES OF

### SAMUEL DAVID GROSS

### A Master in Surgery

His life, which neared the extreme Limits of the Psalmist,
was one unbroken process of Laborious Years.
He filled Chairs in Four Medical Colleges in as many States of the Union,
and added Luster to them all.
He recast Surgical Science as taught in North America,
Formulated anew its Principles,
Enlarged its Domain,
Added to its Art, and imparted fresh Impetus to its Study.
He composed many Books, and among them

### A System of Surgery

Which is read in different tongues, wherever the Healing Art is practiced.
With a Great Intellect, carefully trained and balanced,
He aimed with undivided Zeal
At the Noble End of Lessening Human Suffering
and Lengthening Human Life,
And so rose to the Highest Position yet attained in Science
by any of His Countrymen.
Resolute in Truth, he had no Fear, yet he was
both Tolerant and Charitable.
Living in Enlightened Fellowship with all Laborers in the
World of Science,
He was greatly Honored by the Learned in Foreign Lands
and deeply Loved at Home.

*BEHIND THE VEIL OF THIS LIFE THERE IS A MYSTERY WHICH HE PENETRATED ON THE*

### Sixth Day of May, 1884

HIS MEMORY
Shall Exhort and his Example shall Encourage and Persuade
those who come after him to Emulate Deeds
which, Great in themselves,
Were all Crowned by the Milkwhite Flower of a

### Stainless Life

